# Risk Factors for Tick‐Borne Diseases in Germany: A Scoping Review

**DOI:** 10.1111/zph.70060

**Published:** 2026-04-07

**Authors:** Carolin Schlupp, Matthias Hans Belau

**Affiliations:** ^1^ University Medical Center Hamburg‐Eppendorf Institute of Medical Biometry and Epidemiology Hamburg Germany

**Keywords:** Germany, risk factors, tick bites, tick‐borne diseases, ticks

## Abstract

Tick‐borne diseases (TBDs) have proliferated in Germany. The two most prevalent TBDs, *Lyme‐borreliosis* and tick‐borne encephalitis, can present with nonspecific symptoms and lead to serious neurological complications. To date, a review synthesising the risk factors of acquiring a TBD in Germany is missing. Such a review could mitigate infection risk and support early protection. A scoping review was conducted using the databases MEDLINE/PubMed and Web of Science. Studies on the risk of acquiring TBDs through tick bites in Germany, written in German or English, were included in this review. Two authors screened the papers and charted the results collaboratively. Thirty‐two studies were included in the review, which highlighted the following factors as being associated with an increased risk of tick bites or TBDs: being 3–9 or 50–79 years old; being male; having a higher education level; working outdoors; not having a migration background; visiting a forest kindergarten; spending more time outdoors; not using protective strategies; owning a pet; spring and summer; high humidity and moderate temperatures; unmodified vegetation; high roe deer density; living in southern Germany; and living rurally. This review identified similar risk factors as reviews from other European, Asian and African countries. The findings of this review can be used to improve public health policy, enhance TBD prevention measures, establish a targeted early warning system and enhance clinicians' knowledge.

## Impacts

- The general population will benefit from health practitioners and clinicians' increased knowledge of tick‐borne diseases to better prioritise prevention and early diagnosis, especially by targeting higher risk groups in Germany.
- Through the identification of environmental, temporal and behavioural risk factors, interventions can be targeted to reduce risk, especially in high‐risk areas in Germany.
- This review provides an overview of risk factors and proposes priorities for surveillance and research that can inform a national early‐warning approach.

## 1 Introduction

The proliferation of zoonotic diseases in Germany has been attributed to a combination of factors, including climate change, globalisation and trade (Umweltbundesamt 2023). Zoonoses are defined as pathogens, including bacteria, viruses and parasites, that are transmitted from animals to humans. Currently, approximately 200 zoonoses are known, accounting for most new and emerging human diseases (World Health Organization, Food and Agriculture Organization of the United Nations, and World Organisation for Animal Health 2019). Many of these diseases are transmitted by vectors, living organisms that transfer pathogens from one host to another (Frank et al. 2014). In Germany, *Lyme‐borreliosis* (LB) is the most prevalent tick‐borne zoonosis, primarily transmitted by the most common tick species in Germany, *Ixodes ricinus* (Frank et al. 2014; Fülöp and Poggensee 2008; Umweltbundesamt 2023). This species is also a vector for tick‐borne encephalitis (TBE) (Umweltbundesamt 2023). Another pathogen is bacteria of the order *Rickettsiales*, including *Rickettsia species*, which cause spotted fever group rickettsioses and *Anaplama phagocytophilum*, which causes human granulocytic ehrlichiosis (HGE) and is spread by *Dermacentor reticulatus* and *Haemaphysalis* (Dobler and Wölfel 2009; Glaser et al. 2010).

Ticks require blood meals for moulting between life stages and oviparity (Kahl and Gray 2023). During feeding, ticks remain attached to their hosts for extended periods, which supports pathogen transmission (Kahl and Gray 2023). Most hard ticks, including *Ixodes ricinus* , *Dermacentor reticulatus* and *Haemaphysalis species*, progress through four life stages: egg, larva, nymph and adult, where they are active and seeking hosts during the last three stages (Földvári et al. 2016; Hemmer et al. 2018). They feed for 1% of their lifespan, and otherwise inhabit leaf litter or dense vegetation, as they require warm temperatures and high humidity (Kahl and Gray 2023). Because ticks typically feed on only one host per stage, pathogens must survive through multiple stages to be transmitted, limiting the spread of tick‐borne diseases (TBDs) to areas where established tick populations exist (Pfäffle et al. 2013).

Climate changes in Germany, such as milder winters and earlier higher temperatures, are contributing to increases in tick populations and the emergence of new tick species, including *Hyalomma marginatum* (Hemmer et al. 2018; Umweltbundesamt 2023). Since many TBDs present with non‐specific symptoms and incidences of LB and TBE have been rising since 2002, identifying and understanding the associated risk factors is crucial for public health authorities and healthcare professionals to prevent, recognise and treat the diseases effectively (Dobler and Wölfel 2009; Frank et al. 2014; Glaser et al. 2010; Robert Koch Institut 2025; World Health Organisation 2011). Risk here is the probability that an individual will experience an adverse health outcome and a risk factor is behavioural characteristics, environmental exposures or inherent individual attributes that have been epidemiologically associated with an increased likelihood of developing a given disease (National Library of Medicine 2026a, 2026b).

A comprehensive synthesis of the existing risk factors is crucial for consolidating current knowledge in this domain. Although existing reviews, such as those from Imhoff et al. (2015); Sprong et al. (2018); Pustijanac et al. (2024) and Pfäffle et al. (2013), primarily concentrate on individual pathogens, evaluate risks across broader European contexts, or analyse factors influencing tick abundance (Imhoff et al. 2015; Pfäffle et al. 2013; Pustijanac et al. 2024; Sprong et al. 2018), there remains a notable gap in the literature specific to tick species and pathogens of relevance within Germany.

This deficiency underscores the necessity for the present review, which aims to offer a comprehensive overview of the known epidemiological risk factors associated with human infection with TBDs in Germany.

A scoping review was selected due to its ability to incorporate a wide array of study designs and outcomes, to map key concepts and identify research gaps within an interdisciplinary and dispersed field such as TBD epidemiology. This methodological choice aligns with the overarching goal to improve understanding of strategies to mitigate infection risk and to support early detection.

## 2 Materials and Methods

### 2.1 Search Strategy

The review followed the Preferred Reporting Items for Systematic Reviews and Meta‐Analyses extension for Scoping Reviews (PRISMA‐ScR). We compiled a list of terms and alternative spellings related to ‘risk factor’, ‘tick species’ and ‘tick‐borne disease’, combined the free‐text search strings and added subject headings from the National Library of Medicine. The complete list of terms was combined into a keyword string and entered into both Medline via PubMed and Web of Science on 08/04/2025. Studies were included regardless of publication date, and no filters were applied. More information about the search strategy and the inclusion and exclusion criteria can be found in Table 1.

**TABLE 1:** Search strategy.

Search strategy item	Search strategy detail
Databases searched	MEDLINE/PubMed, Web of Science
Used string of keywords	See Data S1
Applied filters	None
Inclusion criteria	Included risk factors associated with acquiring a tick‐borne disease Locally acquired human infections in Germany Transmission via tick bites Study of multiple individuals (e.g., cross‐sectional, cohort, case–control, etc.) English or German language
Exclusion criteria	Infection occurring outside of Germany, including travel related Risk factors for animal infections Case reports Transmission risk through raw milk Creation of predictive models Laboratory studies on structure and characteristics of pathogens Immunity after vaccination or readiness to receive a vaccine

### 2.2 Study Selection

Both authors screened titles and abstracts based on the inclusion and exclusion criteria. Studies were included if they focused on risk factors associated with acquiring a TBD in Germany, with travel‐related illnesses being excluded.

Studies had to assess the risk of human infection and that transmission had to occur through tick bites, as despite sporadic cases of TBE infection through raw milk, they are rare and are difficult to compare to infections resulting from tick bites (Scarazzato et al. 2024).

Case reports were excluded because they cannot statistically identify risk factors. Lastly, only papers written in English or German were included because these are the researchers' languages and most relevant to the German context. Studies that passed the initial screening were reviewed in full to confirm that all inclusion criteria were met.

### 2.3 Data Extraction

Key data from each of the included studies was charted collaboratively by both reviewers using a data extraction table created for this review. This information included the time period and location of the study, the measured outcome, the size and age group of the study population, the study design and type, the key findings, and, when available, prevalence and risk estimates. Any uncertainties were resolved through discussion and consensus. These data were selected to provide a consistent overview of each study and enable comparison.

## 3 Results

**FIGURE 1:**
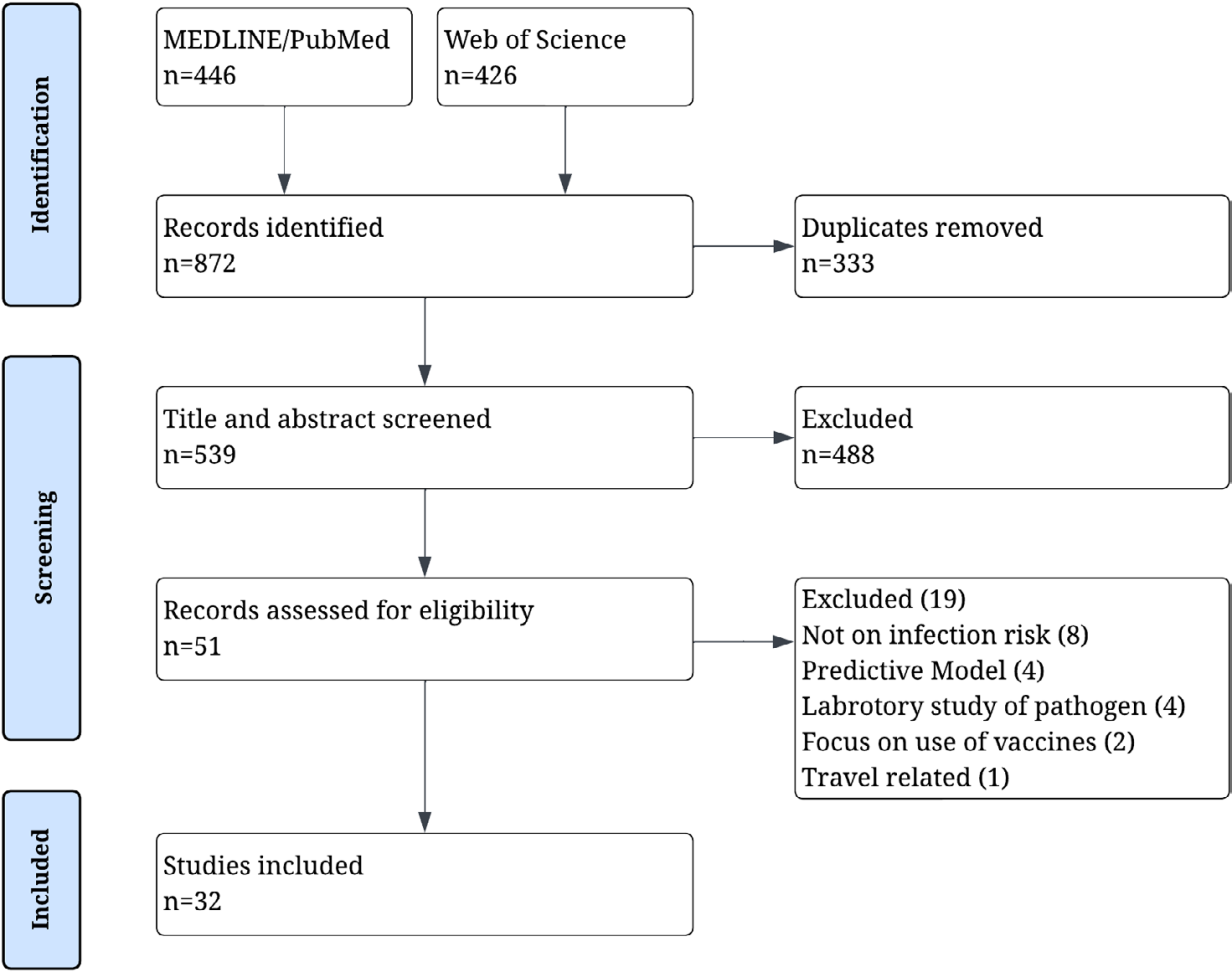
Flow chart of record inclusion.

A total of 872 records were identified, 446 from MEDLINE/PubMed and 426 from Web of Science, with 332 duplicates. The titles and abstracts of the remaining 539 records were screened, causing 488 records to be excluded. Then, the full texts of the remaining 51 records were reviewed, and further 19 studies were excluded for various reasons, as outlined in Figure 1. Ultimately, 32 studies met all criteria and were included in the review.

Of the 32 studies included, six focus on ticks, eighteen focus on LB, five focus on TBE, and three focus on other pathogens. Most of the studies identified seropositive individuals, while others examined tick bites, tick abundance or pathogen prevalence in ticks. The majority of the studies investigated *Ixodes ricinus* . Additionally, the majority of the evidence highlights demographic and behavioural risk factors, followed by climate and environmental factors.

Table 2 provides an overview of the included studies and their key findings, with entomological studies being presented in Table S1. Risk factors are grouped by tick species or TBD and categorised in the text by demographic, living environment, behaviour and practices, climate and weather as well as environment and habitat risk factors.

**TABLE 2:** Results of included studies.

Authors	Study period	Location	Measured outcome	Study population (number and age)	Research design	Key findings	Risk indicator
Schielein et al. 2022	2016–2017 (Sep 2016, Q1 2017, Dec 2017)	Bavaria	Tick bite	3503 adults	Three cross‐sectional studies	85.7% of forestry workers, 73.6% of farmers and 66.3% of outdoor workers had tick bites. Risk was higher living in the environs (OR = 1.98, 95% CI: 1.54–2.55), rural areas (OR = 1.97, 95% CI: 1.49–2.59), and spending 1–3 h outdoors (OR = 1.93, 95% CI: 1.43–2.60).	Occupation, residence, time outdoors
Weisshaar et al. 2006	2004 (Mar–Oct)	Baden‐Wuerttemberg	Tick bites	1707 children (506 forest and 1201 conventional kindergarten)	Cohort study (retrospective and prospective)	Forest kindergarten attendance increased risk of tick bites (RR = 1.87, 95% CI: 1.70–2.07) and LB (RR = 3.56, 95% CI: 1.46–8.66). Preventive measures were more frequent in forest kindergartens, including bug spray (RR = 2.99, 95% CI: 2.37–3.76) and protective clothing (RR = 2.86, 95% CI: 2.51–3.27).	Forest kindergarten, preventive measures
Sammito et al. 2019	2008–2009 (Oct–Sep) & 2012–2014 (Dec–Sep)	Northern Germany	Tick Bite from *Ixodes ricinus*	1156 military personnel	Cross‐sectional	Most tick bites occurred May–September (88%). Incidence was 0.31 bites/1000 h in training April–November.	Season
Böhm et al. 2023	2003–2006 & 2014–2017	Germany	Lyme‐borreliosis Seroprevalence	11,626 children (baseline) and 15,023 (Wave 2)	Two cross‐sectional studies	Compared to 3–6‐year‐olds, 14–17‐year‐olds had higher odds of LB (Wave 1: OR = 2.77, 95% CI: 1.94–3.97; Wave 2: OR = 5.35, 95% CI: 2.10–13.64). Males also had higher odds (Wave 1: OR = 1.51, 95% CI: 1.24–1.83; Wave 2: OR = 2.01, 95% CI: 1.28–3.15). Reginal risk was elevated in Bavaria (Wave 1: OR = 1.94, 95% CI: 1.28–2.93) and the South‐East (Wave 2: OR = 2.66, 95% CI: 1.09–6.51). Rural residence (OR = 1.61, 95% CI: 1.11–2.35), exercising 3–5 times/week (Wave 1: OR = 2.1, 95% CI: 1.24–3.55) and no migration background (OR = 3.76, 95% CI: 2.53–5.59) increased risk. Pet ownership (OR = 1.34, 95% CI: 1.09–1.63) was a risk factor only in univariate analysis. Low SES reduced risk (Wave 1: OR = 0.50, 95% CI: 0.32–0.79; Wave 2: OR = 0.35, 95% CI: 0.15–0.84).	Age, no low SES, no migration background, pet ownership, physical activity, sex, region, residence
Böhm et al. 2025	2019 (Jun–Aug)	Bavaria	Lyme‐borreliosis Seroprevalence or diagnosis	377 infected individuals (77 children)	Cross‐sectional	Most were female (58.4%), aged 35–59 (33.7%) or 60–79 (34.2%), rural residents (63.8%), and with middle (44.9%) or high (45.2%) education. Risk increased with more days outdoors and tick‐exposed occupations.	Age, education, time outdoors, occupation, residence, sex
Böhmer et al. 2021	2013–2020 (Mar–Dec)	Bavaria	Lyme‐Borreliosis diagnosis	35,458 LB cases	Ecological study	Incidence peaked in ages 5–9 and 60–69, was higher in males, and 58.6% occurred June–August.	Age, season, sex
Coors et al. 2022	2018–2020 (Feb–Feb)	Bonn	Lyme‐borreliosis Seroprevalence	2865 participants aged ≥ 30	Cohort study	Risk increased per year of age (OR = 1.03, 95% CI: 1.02–1.05), for males (OR = 1.65, 95% CI: 1.01–2.73) and highly educated individuals (OR = 1.83, 95% CI: 1.10–3.14).	Age, education, sex
Dehnert et al. 2012	2003–2006	Germany	Lyme‐borreliosis Seroprevalence	12,614 participants aged 1–17	Cross‐sectional study	Males had higher odds of antibodies (OR = 1.35, 95% CI: 1.10–1.66). Risk increased with age (females: OR = 1.07, 95% CI: 1.03–1.11; males: OR = 1.13, 95% CI: 1.09–1.16) per year. Higher risk was associated with living south (OR = 1.34, 95% CI: 1.03–1.75) and cat ownership (OR = 1.50, 95% CI: 1.19–1.90). Migration background reduced risk (OR = 0.28, 95% CI: 0.19–0.42).	Age, no migration background, pet ownership, region, sex
Faulde et al. 2014	2009 (Apr–Sep)	Northwestern Germany	Lyme‐borreliosis Seroprevalence	566 military personnel	Cohort study	Military personnel had an LB incidence of 176/10,000 compared to the clinical rate (70.7/10,000). 99% occurred June–September.	Occupation, season
Franke et al. 2008	Not reported	Thuringia	Lyme‐borreliosis diagnosis via PCR	618 *Ixodes ricinus* ticks	Cross‐sectional study	6.1% ticks tested positive for LB. with peak prevalence in fall (10%), followed by summer (6.5%) and spring (4.0%).	Season
Hassenstein et al. 2023	2014–2019	Augsburg, Berlin, Münster and Hanover	Lyme‐borreliosis Seroprevalence	14,207 participants aged 20–74	Cross‐sectional study	IgG seropositivity increased by 1.26 (95% CI: 1.16–1.38) per 10 years of age. Males had higher odds (OR = 2.54, 95% CI: 2.05–3.13) and migration background reduced the risk (OR = 0.56, 95% CI: 0.41–0.78).	Age, no migration background, sex
Jurke et al. 2015	2011–2013	North Rhine‐Westphalia	Lyme‐borreliosis Seroprevalence	722 forestry employees aged 18–66	Cross‐sectional	Seropositive participants were more likely to be male (OR = 5.28, 95% CI: 2.39–11.67), older than 50 (OR = 2.02, 95% CI: 1.14–3.58), work outdoors (OR = 2.54, 95% CI: 1.45–4.46), and have 50 bites or more (OR = 2.04, 95% CI: 1.39–2.99).	Age, occupation, sex, tick bites
Mehnert and Krause 2005	2002–2003	Eastern Germany	Lyme‐borreliosis Seroprevalence	6987 cases; 3019 (2002) and 3968 (2003)	Ecological study	Most LB cases were reported in Brandenburg or Saxony (81%) and incidences peaked at 5–9‐years (2002: 18.3/100,000, 2003: 28.9/100,000) and 60–64 (2002: 39.2/100,000) and 65–69 (2003: 48.6/100,000). Most were females (55%) and occurred June–September (70%), peaking in July.	Age, region, season, sex
Nübling et al. 1999	Not reported	Southwestern Germany	Lyme‐borreliosis Seroprevalence	792 participants	Cross‐sectional study	Compare to individuals with no outdoor leisure time the risk increased fourfold for agriculture workers and sevenfold for forestry workers. Risk increased with age across all groups.	Age, occupation
Rath et al. 1996	1992 (Feb–Sep)	Brandenburg	Lyme‐borreliosis Seroprevalence	630 forestry workers and 200 control	Cross‐sectional study	Forestry workers had a significantly higher IgG seropositivity rate (8%) compared to controls (4%). Tick bite frequency increased with age and years of employment, 6.4% (0–1 years) to 63.4% (> 10 years).	Age, occupation
Skufca et al. 2023	2016–2020 (Jan–Dec)	Germany	Lyme‐borreliosis Seroprevalence or diagnosis	63,940 LB cases	Ecological study	Incidence was higher in females (39.9/100,000, 95% CI: 39.1–40.7) than males (33.5/100,000, 95% CI: 32.8–34.2). Peaks occurred in children (5–9 years) and adults (55–74 years). Most (68%) were in June–September, peaking in June/July.	Age, season, sex
von Wissmann et al. 2015	2010–2011 (Jul–Aug)	Bavaria	Tick bites	107 participants	Cross‐sectional study	Lower risk of LB in females and a higher risk in older individuals and those aged 40–65, but all not significant. Gardeners had reduced risk for LB.	Occupation, time outdoors
Wilking and Stark 2014	2009–2012 (Jan–Dec)	Eastern Germany	Lyme‐borreliosis Seroprevalence or diagnosis	18,894 reported	Ecological study	Incidence peaked for ages 5–9 and 50–69 years. Most were female (55.3%). 60% of all cases occurred June–September, peaking in July. Most cases were in Brandenburg, Mecklenburg‐Western Pomerania, and Saxony.	Age, region, season, sex
Wilking et al. 2015	2008–2011	Germany	Lyme‐borreliosis Seroprevalence	6945 participants aged 18–79	Cross‐sectional study	Seroprevalence was higher among males, individuals > 59, rural residents, and people in southern Germany. Non‐German citizenship reduced risk.	Age, not German citizenship, region, residence, sex
Woudenberg et al. 2020	1997–1999 & 2008–2011	Germany	Lyme‐borreliosis Seroprevalence	5510 participants (1997–1999) & 6957 participants (2008–2011); aged 18–79	Two cross‐sectional studies	70–79 years, increased risk of seropositivity, as did being male and residing in southern Germany. Rural residence increased the risk for seroconversion.	Age, region, residence, sex
Borde et al. 2019	2001–2018	Germany	Tick‐borne encephalitis Seroprevalence	6073 TBE cases	Ecological study	More cases were male (63.5%). Infections peaked June–July, and onset of infections shifted earlier, by 0.69 days/year.	Season, sex, year
Friedsam et al. 2022	2018–2020 (Jan–Dec)	Bavaria and Baden‐Wuerttemberg	Tick‐borne encephalitis PCR or Seroprevalence	581 TBE cases	Cohort study	More infections occurred in Baden‐Württemberg (53%) than Bavaria. Areas with more TBE cases were warmer in summer and predominantly covered in coniferous forest (59%) or homogenous grassland (23%). Odds increased with summer precipitation (OR = 2.8), annual frost days (OR = 2.3) and population density (OR = 1.8). Negative associations were found with tick density (OR = 0.8) and snow cover days (OR = 0.5).	Region, season, vegetation
Hellenbrand et al. 2019	2001–2018	Germany	Tick‐borne encephalitis PCR or Seroprevalence	6063 TBE cases	Ecological study	89.0% of cases occurred in Baden‐Württemberg and Bavaria. Most cases occurred May–October (91.1%), peaking in June–July (49.4%). Incidence was higher among males (63.5%) and 40–69‐year‐olds. Vaccine uptake was higher in regions with elevated TBE risk.	Age, region, season, sex
Kiffner et al. 2010	2001–2008	Germany	Tick‐borne encephalitis PCR or Seroprevalence	TBEV cases	Ecological study	TBE infection probability increased with coniferous forest cover. Roe deer density showed positive correlation, whereas red fox and red deer densities showed negative correlations. A temperature increase in spring was negatively associated with TBE incidence.	Season, vegetation, wild animal density
Nygren et al. 2022	2018–2020 (Jan–Dec)	South Germany	Tick‐borne encephalitis PCR or Seroprevalence	581 TBE cases and 975 controls	Case–Control study	Controls had higher proportion of vaccinated individuals (24%) compared to cases (3%). Several risk factors included: living rurally (OR = 1.32, 95% CI: 1.04–1.67), dog ownership (OR = 2.45, 95% CI: 1.85–3.24), straying from paths (OR = 1.82, 95% CI: 1.26–2.63) and gardening ≥ 4 times/week (OR = 1.83, 95% CI: 1.11–3.02). Compared to ≤ 1 walks/week, the risk increased for 1–3 walks/week (OR = 1.44, 95% CI: 1.03–2.01) and for ≥ 4 walks/week (OR = 2.11, 95% CI: 1.42–3.12). Compared to outdoor activity 1/week or less, 1–3 times/week resulted in increased risk (OR = 1.66, 95% CI: 1.18–2.33), and ≥ 4 times/week (OR = 1.64, 95% CI: 1.10–2.43). Compared to gardens more than 1 km from a forest, gardens located 500–250 m away had an increased risk (OR = 1.60, 95% CI: 1.13–2.24), and those < 250 m (OR = 2.54, 95% CI: 1.82–3.56). Protective factors included raw milk intake (OR = 0.49, 95% CI: 0.37–0.64), keeping lawns mown (OR = 0.63, 95% CI: 0.43–0.91), and using multiple protective strategies, 2–4 strategies (OR = 0.52, 95% CI: 0.40–0.68) or 5–7 strategies (OR = 0.34, 95% CI: 0.23–0.51), compared to 0–1 strategies.	Pet ownership, no protective strategies, residence, time outdoors, no vaccine
Wölfel et al. 2017	2008 (May–Jun)	Brandenburg	Rickettsiales Seroprevalence	559 forestry workers	Cross‐sectional	27.5% of forestry workers were seropositive, males more so. Males had higher odds of being bitten (OR = 2.53, 95% CI: 1.22–5.25), as did those over 55 years (OR = 3.41, 95% CI: 1.10–10.70).	Age, occupation, sex
Fingerle et al. 1997	1983–1984	South Germany	Human Granulocytic Ehrlichiosis Seroprevalence	150 forestry workers, 105 LB patients and 103 controls	Cross‐sectional study	Forestry workers (14%) and LB patients (11.4%) had higher HGE rates than the control group (1.9%).	Occupation

### 3.1 Demographic Factors

#### 3.1.1 Age

Young children, aged between 3 and 9 years (Hauck et al. 2020; Robert Koch Institut 2025; Sammito et al. 2019; Scarazzato et al. 2024) and older adults, aged between 50 and 79 years (Böhm et al. 2025; Böhmer et al. 2021; Jurke et al. 2015; Mehnert and Krause 2005; Skufca et al. 2023; Wilking et al. 2015; Wilking and Stark 2014; Wölfel et al. 2017; Woudenberg et al. 2020), were identified as having the greatest risk for LB, TBE and Rickettsiales. Furthermore, the risk of LB infection increases with age (Coors et al. 2022; Dehnert et al. 2012; Hassenstein et al. 2023; Nübling et al. 1999; Rath et al. 1996).

#### 3.1.2 Sex

Males have increased odds of getting a tick bite (Wölfel et al. 2017) and also have a higher incidence of TBE (63.5%) than females (Borde et al. 2019; Hellenbrand et al. 2019). There were 11 studies that found a significant effect of sex on LB risk, eight suggest that males have higher risk (Böhm et al. 2023; Böhmer et al. 2021; Coors et al. 2022; Dehnert et al. 2012; Hassenstein et al. 2023; Jurke et al. 2015; Wilking et al. 2015; Woudenberg et al. 2020) and four that females have higher risk (Böhm et al. 2025; Mehnert and Krause 2005; Skufca et al. 2023; Wilking and Stark 2014).

#### 3.1.3 Education and Socioeconomic Status

Two studies on LB found that a higher level of education increased odds of infection (Böhm et al. 2025; Coors et al. 2022). A separate study on LB reported that having a low socioeconomic status (SES) was a protective factor (Böhm et al. 2023).

#### 3.1.4 Occupation

Forestry workers, farmers and other outdoor workers have a higher prevalence of tick bites (Schielein et al. 2022). Six studies found an increased risk of LB for military personnel (Faulde et al. 2014), forestry workers (Nübling et al. 1999; Rath et al. 1996), agricultural workers (Nübling et al. 1999) and individuals working outdoors in general (Böhm et al. 2025; Jurke et al. 2015). However, gardeners had a slightly reduced risk (von Wissmann et al. 2015). Forestry workers and hunters are at greater risk of contracting *A. phagocytophilum* (Fingerle et al. 1997; von Wissmann et al. 2015; Wölfel et al. 2017) and forestry workers of contracting *Rickettsia* spp. (Jurke et al. 2015). Furthermore, among forestry workers, receiving 50 or more tick bites over the course of their careers was associated with double the odds of contracting LB (Jurke et al. 2015).

#### 3.1.5 Migration Background and Citizenship

Three studies found that having a migration background was associated with a reduced risk of contracting LB (Böhm et al. 2023; Dehnert et al. 2012; Hassenstein et al. 2023). One study found that not having German citizenship was associated with a lower likelihood of becoming infected with LB (Wilking et al. 2015).

### 3.2 Living Environment, Behaviour and Practices

#### 3.2.1 Forest Kindergarten

Attending a forest kindergarten also increases the risk of getting at least one tick bite (Weisshaar et al. 2006) and was associated with a more than threefold risk of LB (Schielein et al. 2022).

#### 3.2.2 Time Outdoors

Spending one to 3 h outdoors almost doubles the odds of getting a tick bite (Schielein et al. 2022), and spending more time outdoors increases the risk for getting LB (Böhm et al. 2025). More time spent gardening, walking outside and participating in other outdoor activities also increased the odds of infection with TBE (Nygren et al. 2022). However, most studies did not differentiate which outdoor spaces increased exposure risk.

#### 3.2.3 Physical Activity

According to a study using German Health Interview and Examination Survey for Children and Adolescents (KiGGS) data, higher physical activity levels were associated with increased risk of infection with LB (Böhm et al. 2023).

#### 3.2.4 Protective Strategies

In a study on TBE, 24% of controls were fully vaccinated compared to 3% of cases (Nygren et al. 2022). Other protective measures, like lawn mowing, using multiple protective strategies and consuming raw milk were associated with reduced TBE risk (Nygren et al. 2022).

#### 3.2.5 Pet Ownership

Two studies found that owning a pet (Böhm et al. 2023), more specifically a cat (Böhm et al. 2023; Dehnert et al. 2012), increased the odds of an LB infection. Another study found that owning a dog more than doubled the odds of contracting TBE (Nygren et al. 2022). However, two other studies found no association between pet ownership and LB risk (Hassenstein et al. 2023; Wilking et al. 2015).

### 3.3 Climate and Weather

#### 3.3.1 Season and Temporal Factor

*Ixodes ricinus* activity peaks in May and June (Gethmann et al. 2020; Hauck et al. 2020; Raileanu et al. 2022), while *D. reticulatus* activity peaks in May and *H. concinna* in June (Raileanu et al. 2022). The number of cases of LB and TBE peaks in July, with cases primarily occurring between June and September (Böhmer et al. 2021; Borde et al. 2019; Faulde et al. 2014; Hellenbrand et al. 2019; Mehnert and Krause 2005; Skufca et al. 2023; Wilking and Stark 2014). Peaks in *A. phagocytophilum* were also observed in July, while *Rickettsia* spp. peaked between April and June (Blazejak et al. 2017). One study found that the onset of TBE infections shifted earlier by 0.69 days per year from 2001 to 2018 (Borde et al. 2019). Additionally, the prevalence of *Rickettsia* spp. increased from 2005 to 2015 (Blazejak et al. 2017).

#### 3.3.2 Humidity

According to one study, high relative humidity is associated with an increased abundance of *Ixodes ricinus* (Gethmann et al. 2020). Furthermore, the risk of TBE infection increases with greater summer precipitation (Friedsam et al. 2022). However, another study found no effect of relative humidity on the abundance of *Ixodes ricinus* (Hauck et al. 2020).

#### 3.3.3 Temperature

One study found that milder temperatures were associated with an increase in the number of *Ixodes ricinus* ticks present (Gethmann et al. 2020), while another study found no such effect of temperature (Kiffner et al. 2010). Furthermore, higher spring temperatures were associated with decreased TBE incidence (Kiffner et al. 2010) and TBE risk increased with fewer annual frost days while it decreased with more snow cover days (Friedsam et al. 2022).

### 3.4 Environment and Habitat Factors

#### 3.4.1 Vegetation

Tick abundance was found to be highest in areas with thick, unmodified vegetation (Richter and Matuschka 2011), and high populations of *Ixodes ricinus* were found in forests (Gethmann et al. 2020; Hauck et al. 2020). Dense forest cover, was associated with a higher probability of TBE infection (Friedsam et al. 2022; Kiffner et al. 2010). Similarly, higher prevalence of *Rickettsia* spp. and *A. phagocytophilum* were detected in the ‘Misburger Forest’ compared to another forest with similar vegetation (Blazejak et al. 2017).

#### 3.4.2 Wild Animal Density

The probability of TBE infection showed a positive association with roe deer population density and a negative association with red fox and red deer population densities (Kiffner et al. 2010).

#### 3.4.3 Region

**FIGURE 2:**
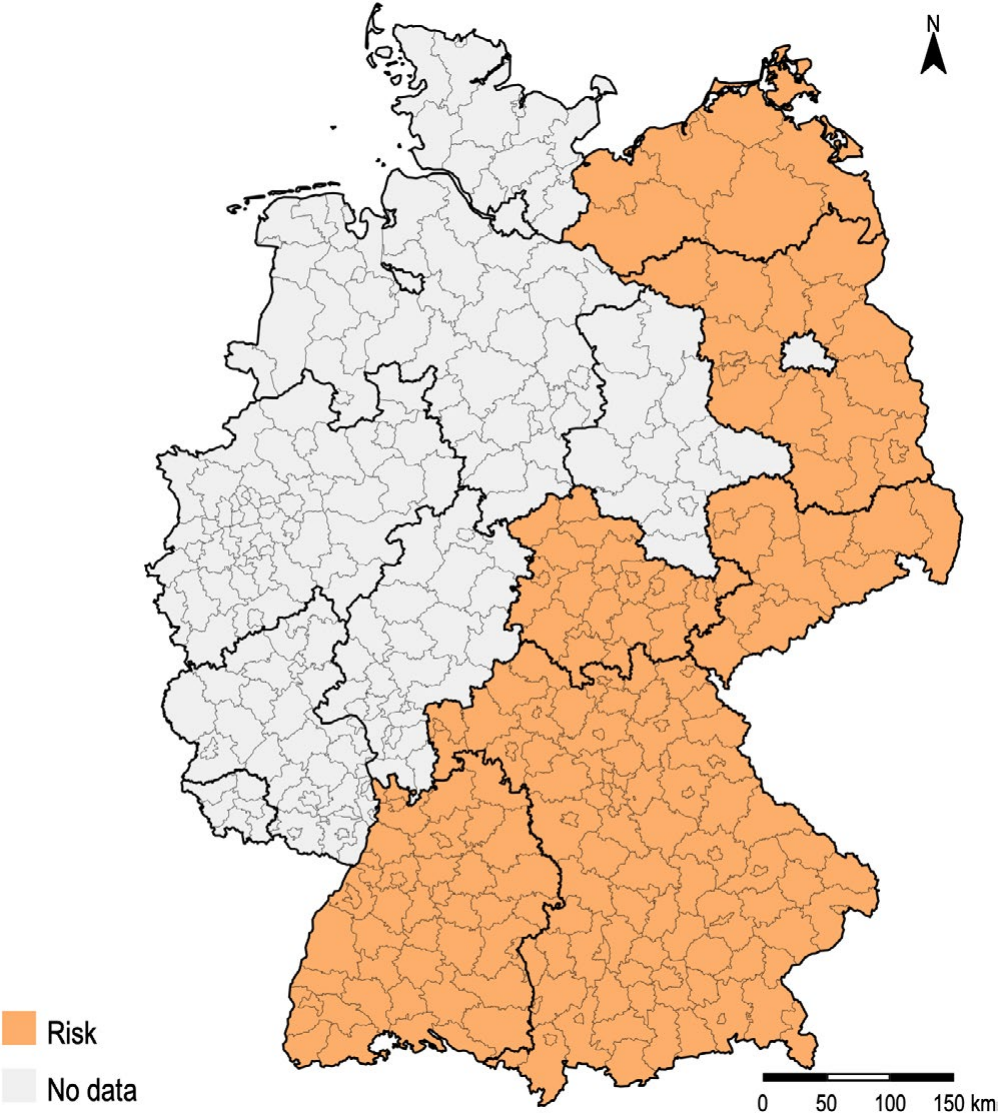
LB and TBE risk regions.

Southern Germany was identified as an LB risk region in three studies (Dehnert et al. 2012; Wilking et al. 2015; Wilking and Stark 2014) and southeastern Germany in one study (Böhm et al. 2023). Several states in the south showed an increased risk of LB, including Bavaria (Böhm et al. 2023; Woudenberg et al. 2020), Baden‐Württemberg (Woudenberg et al. 2020), Thuringia (Woudenberg et al. 2020), Saxony (Böhm et al. 2023; Sammito et al. 2019; Scarazzato et al. 2024). An elevated risk was reported in Brandenburg and Mecklenburg‐Western Pomerania (Wilking and Stark 2014). Most infections with TBE occurred in Baden‐Württemberg and Bavaria (Hellenbrand et al. 2019), with slightly more occurring in Baden‐Württemberg than in Bavaria (Friedsam et al. 2022). Figure 2 displays the regions at risk for LB and TBE.

#### 3.4.4 Residence

Having a residence in rural areas nearly doubles one's odds of getting a tick bite (Schielein et al. 2022). Four studies found that living in a rural area increases the risk of LB (Böhm et al. 2023, 2025; Wilking et al. 2015; Woudenberg et al. 2020), with one analysis reporting an odds ratio of 1.61 (95% CI: 1.11–2.35) (Böhm et al. 2023). Living in a rural area also increases the odds of infection with TBE (Nygren et al. 2022).

## 4 Discussion

The aim of this scoping review was to identify risk factors for humans contracting TBDs in Germany. The majority of the findings align with the current knowledge of risk factors for TBDs in other European, Asian, and African countries (Burn et al. 2023; Ergünay et al. 2020; Fischhoff et al. 2019; Hansford et al. 2022; Jaenson et al. 2012; Nasirian 2019; Richardson et al. 2019).

Several identified factors relate to vulnerability and coping capacity, including demographic characteristics and preventive behaviours. Consistent with other reviews, children (< 5 years) and adults (50–70 years) had the highest risk of TBDs, likely due to greater outdoor exposure (Burn et al. 2023; Fischhoff et al. 2019). Most findings suggest males are at higher risk, consistent with other findings across Europe (Burn et al. 2023). Males likely have a higher risk as many male dominated professions are done outdoors, which increases their exposure. Higher education and SES also coincided with higher risk. Likewise, in a review on Crimean‐Congo hemorrhagic fever (CCHF) high knowledge about CCHF and high literacy level were risk factors for infection (Nasirian 2019). This could be explained by individuals with higher education and higher SES having more time to spend outdoors. This could also explain occupations involving extended outdoor activity, such as forestry workers, military personnel, farmers and hunters having an increased risk (Burn et al. 2023; Nasirian 2019). Lastly, migration background and non‐German citizenship were associated with a decreased risk of contracting LB, with another European review finding that being Caucasian coincided with an increased risk of acquiring a TBD (Fischhoff et al. 2019). Especially considering that studies in the United States have found white persons to have higher incidences and prevalences of LB but minority groups to report disease onset in the fall and have more disseminated manifestations of LB, these factors should be further studied (Gould et al. 2024). Preventative measures such as being vaccinated against TBE and the use of other personal protective measures decreased TBE risk, which is supported by other reviews finding the use of personal protective measures such as wearing insect repellent, protective clothing, being vaccinated and being informed on the risks to be protective against TBD (Nasirian 2019; Richardson et al. 2019). Therefore, the use of protective measures is an important measure to reduce the risk of acquiring a TBD.

In addition to vulnerability, several factors influence the level of exposure to ticks. This review found forest kindergartens to be a risk factor for contracting a TBD. No other reviews were found to list this as a risk factor, which could be due to forest kindergartens not being as widespread in other parts of the world or a difference in exposures included in existing studies. Spending more time outdoors was also linked to an increased risk of getting a tick bite, LB, or TBE, especially time spent gardening or walking outside. Other reviews did not study this exact outcome, but Fischhoff et al. (2019) found that having a garden increased risk, and Nasirian (2019) found that spending more time in rural areas increased risk (Fischhoff et al. 2019; Nasirian 2019). Time outdoors might increase risk as it increases exposure to ticks and therefore TBDs. While specific forms of physical activity, such as hiking have been found to increase the risk of LB, this review found that general physical activity leads to an increase (Nygren et al. 2022). This should however be further researched to determine which kinds of physical activity increase the risk for LB and how to protect individuals during those activities as to not deter physical activity in the population. Pet ownership was found to be a risk factor and likely is as other reviews found similar results (Fischhoff et al. 2019; Nasirian 2019). Interestedly, cat ownership is associated with an increased risk, which could possibly be linked to outdoor cats being exposed to ticks, but studies did not specify if the risk was only increased for outdoor cats so it could also point towards the presence of a confounder.

Hazard related factors refer to environmental and climatic conditions that influence the presence, abundance and infection prevalence of ticks. Climatic factors also showed an effect on the risk of contracting a TBD. Findings suggest spring and summer to be peak times for tick activity (Jaenson et al. 2012). This is likely because humidity level and temperature were also found to be risk factors. In higher humidity, ticks climb higher in vegetation, making them able to infect larger mammals, including humans (Jaenson et al. 2012). Jaenson et al. similarly found tick activity to begin in spring with mild temperatures of approximately 5 to 10 degrees Celsius but found that a cold winter followed by a fast increase in temperature was optimal (Jaenson et al. 2012). Mild temperatures in the spring seem to be consistently associated with tick activity within Europe, but the influence of winter temperatures remains unclear. Most climate and weather factors found in this review seem consistent with other research, especially in Europe. Environmental factors affected the risk of getting infected with a TBD. One major risk factor for TBDs, likely due to higher tick densities, was unmodified dense vegetation, primarily coniferous forests or grasslands (Fischhoff et al. 2019; Nasirian 2019). This is likely due to ticks needing high humidity and moderate temperature to survive, which could make dense vegetation more suitable for tick survival (Kahl and Gray 2023). Furthermore, Roe deer are an important host for ticks, and their increase in population density has been found to be an influencing factor (Jaenson et al. 2012). Fischhoff et al. (2019) found an abundance of all deer increased risk for TBD, while this review found the red deer population to be negatively associated with risk, possibly due to different distributions of deer types in the different studies. One review found that most studies on ticks were from central Europe, including Germany, but some locations in Germany did not have any ticks present (Hansford et al. 2022). This could be due to states in southern Germany having warmer climate conditions. Places with lower human density and living rurally were associated with an increased risk of infection with TBDs (Fischhoff et al. 2019; Nasirian 2019). Possibly, rural areas have more unaltered land and vegetation, leading to higher tick abundances than in the city.

Most of the findings from around the world were consistently found to be risk factors in Germany. However, Richardson et al. (2019) found some further behavioural risks, such as having a bird feeder and having log piles, but could also be relevant risks in Germany (Richardson et al. 2019). Furthermore, Fischhoff et al. found that a higher nymph density was associated with a higher risk of infection. Comparatively, other studies found total tick density to be associated with a higher risk of contracting a TBD. Hansford et al. (2022) found that in addition to forests, large tick populations were reported in parks, graveyards, fields and botanical gardens (Hansford et al. 2022). These are also large green spaces in which some areas of vegetation can go undisturbed.

The consistency of the results of this review with other reviews in Europe and other parts of the world is positive. However, there are some limitations that should be considered to contextualise the results. Language limitations and gaps in database coverage could result in some relevant studies being missed. Data charting is subjective, which could cause a reporting bias. Furthermore, as with most scoping reviews, no critical quality appraisal of included studies was conducted, meaning that studies may vary in methodological quality. Included studies were heterogeneous in design, population and outcome measures. Most assessed human incidence of LB or TBE, while others used proxies such as tick bites or tick abundance, limiting comparability. As no meta‐analysis was conducted, there can be no statement made on the precise effect estimates. Further limitations include the exclusion of case reports, travel‐related infections and non‐tick bite transmissions, as this could exclude rare risk factors that are hard to track. The focus on Germany could cause some risks of neighbouring countries to be missed. In addition, climate factors appeared more frequently associated with TBE than LB. However, this difference could reflect variation in study focus or surveillance practices, rather than a difference in climate sensitivity, as seasonal patterns were also observed for LB. A strength is the use of two authors to review and chart data, as it aims to reduce bias. Considering the lack of overarching data on risk factors for TBDs in Germany, this review provides an overview of existing literature, which was missing prior.

Considering the general limitations of a scoping review, evidence found and summarised should be used in future research. More studies should be conducted for risk factors of TBDs in general to provide more data for a future systematic literature review and meta‐analysis. Furthermore, new and emerging TBDs, such as CCHF or Rickettsiosis, in Germany should be further studied to identify if similar risk factors are applicable to these diseases. More research into this topic allows public health measures to be developed more effectively.

## 5 Conclusions

This review identified risk factors for contracting a TBDs in Germany. These findings are important for public health policymakers, specialists and clinicians. Some of these factors, such as education level, occupation and vegetation, can be addressed through public health policy. Examples include increasing awareness of TBD risk and preventive measures to use during outdoor activities as well as providing protections for certain occupations, and using targeted environmental management, including localised vegetation control or limited acaricide application, to reduce human‐tick contact. These findings can inform targeted risk communication and protective measures. They can also use the data to create risk assessments and targeted early warning systems for TBDs in Germany. Such a system would benefit clinicians because they could use it to evaluate the likelihood that their patients have a TBD, as many TBDs have nonspecific symptoms.

## Author Contributions

**C.S., M.H.B.:** conceptualisation; **C.S., M.H.B.:** methodology; MHB: project administration; **M.H.B.:** supervision; **C.S., M.H.B.:** data extraction; **C.S., M.H.B.:** formal analysis; **C.S.:** writing – original draft; **C.S., M.H.B.:** writing – review and editing.

## Funding

This study was supported by the Early warning system for assessing epidemic threats in Germany (FRED) project. The FRED project itself was funded by the German Federal Ministry of Research, Technology and Space (BMFTR – https://www.bmftr.bund.de) under grant number 03DPS1021 to MHB. MHB and CS received financial support. The funders had no role in study design, data collection and analysis, decision to publish, or preparation of the manuscript.

## Ethics Statement

The authors have nothing to report.

## Consent

The authors have nothing to report.

## Conflicts of Interest

The authors declare no conflicts of interest.

## Supporting information


**Data S1:** zph70060‐sup‐0001‐Supinfo1.docx.

**Table S1:** Results of included entomological studies.

## Data Availability

All relevant data are included in the paper or its Supporting Information.
