# Supplementary material for: Risk Factors for Tick‐Borne Diseases in Germany: A Scoping Review
Source: Zoonoses Public Health. 2026 Apr 7;73(4):297–313. doi: 10.1111/zph.70060 (PMC13144437; doi:10.1111/zph.70060)
Supplement: Supplementary file 1 — Data S1: zph70060‐sup‐0001‐Supinfo1.docx. [file ZPH-73-297-s002.docx]

**Supplementary Material S1**

**String of keywords in Medline**:

((((risk* or risk factor* or Predictor* or Determina* or Barrier* or Facilitator* or Cause* or Determinant).ab. or (risk* or risk factor* or Predictor* or Determina* or Barrier* or Facilitator* or Cause* or Determinant).ti.) OR ((Risk or Risk Factors or Forcasting or Root Cause Analysis or Epidemiologic Factors).sh.)) AND (((tick* or tick-borne or tickborne or tick bite* or tick lifecycle* or anaplasmosis or babesiosis or Bourbon virus or colorado tick fever or tick fever or ehrlichiosis or hard tick relapsing fever or heartland fever or lyme disease or powassan virus or rickettsiosis or rocky mountain spotted fever or soft tick relapsing fever or STARI or southern tick-associated rash illness or tularemia or crimean-congo hemorrhagic fever or viral hemorrhagic fever* or CCHF or CCHFV or argas reflexus or carios vespertilionis or Dermacentor or haemaphysalis or hyalomma or ixodes or rhipicephalus sanguieus).ab. or (tick* or tick-borne or tickborne or tick bite* or tick lifecycle* or anaplasmosis or babesiosis or Bourbon virus or colorado tick fever or tick fever or ehrlichiosis or hard tick relapsing fever or heartland fever or lyme disease or powassan virus or rickettsiosis or rocky mountain spotted fever or soft tick relapsing fever or STARI or southern tick-associated rash illness or tularemia or crimean-congo hemorrhagic fever or viral hemorrhagic fever* or CCHF or CCHFV or argas reflexus or carios vespertilionis or Dermacentor or haemaphysalis or hyalomma or ixodes or rhipicephalus sanguieus).ti.) OR ((Ticks or Tick-Borne Diseases or Tick Bites or Anaplasmosis or Babesiosis or Colorado Tick Fever or Colorado Tick Fever Virus or Ehrlichiosis or Relapsing Fever or Ixodidae or Lyme Disease or Encephalitis Viruses, Tick-Borne or Encephalitis, Tick-Borne or Rickettsia Infections or rocky mountain spotted fever or Ornithodoros or Argasidae or Tularemia or Hemorrhagic Fever, Crimean or Hemorrhagic Fevers, Viral or Argas or Dermacentor or Haemaphysalis longicornis or Ixodes or Rhipicephalus sanguineus or Ornithodoros or Spotted Fever Group Rickettsiosis or Amblyomma).sh.)) AND (Germany.ab. or Germany.ti. OR (germany).sh.))

**String of keywords in Web of Science**:

((AB=(risk* or risk factor* or Predictor* or Determina* or Barrier* or Facilitator* or Cause* or Determinant)) OR TI=(risk* or risk factor* or Predictor* or Determina* or Barrier* or Facilitator* or Cause* or Determinant) OR AB=(Risk or Risk Factors or Forcasting or Root Cause Analysis or Epidemiologic Factors) OR TI=(Risk or Risk Factors or Forcasting or Root Cause Analysis or Epidemiologic Factors)) AND ((AB=(tick* OR tick-borne OR tickborne OR tick bite* OR tick lifecycle* OR anaplasmosis OR babesiosis OR Bourbon virus OR colorado tick fever OR tick fever OR ehrlichiosis OR hard tick relapsing fever OR heartland fever OR lyme disease OR powassan virus OR rickettsiosis OR rocky mountain spotted fever OR soft tick relapsing fever OR STARI OR southern tick-associated rash illness OR tularemia OR crimean-congo hemorrhagic fever OR viral hemorrhagic fever* OR CCHF OR CCHFV OR argas reflexus OR carios vespertilionis OR Dermacentor OR haemaphysalis OR hyalomma OR ixodes OR rhipicephalus sanguieus) OR TI=(tick* OR tick-borne OR tickborne OR tick bite* OR tick lifecycle* OR anaplasmosis OR babesiosis OR Bourbon virus OR colorado tick fever OR tick fever OR ehrlichiosis OR hard tick relapsing fever OR heartland fever OR lyme disease OR powassan virus OR rickettsiosis OR rocky mountain spotted fever OR soft tick relapsing fever OR STARI OR southern tick-associated rash illness OR tularemia OR crimean-congo hemorrhagic fever OR viral hemorrhagic fever* OR CCHF OR CCHFV OR argas reflexus OR carios vespertilionis OR Dermacentor OR haemaphysalis OR hyalomma OR ixodes OR rhipicephalus sanguieus) OR AB=(Ticks or Tick-Borne Diseases or Tick Bites or Anaplasmosis or Babesiosis or Colorado Tick Fever or Colorado Tick Fever Virus or Ehrlichiosis or Relapsing Fever or Ixodidae or Lyme Disease or Encephalitis Viruses, Tick-Borne or Encephalitis, Tick-Borne or Rickettsia Infections or rocky mountain spotted fever or Ornithodoros or Argasidae or Tularemia or Hemorrhagic Fever, Crimean or Hemorrhagic Fevers, Viral or Argas or Dermacentor or Haemaphysalis longicornis or Ixodes or Rhipicephalus sanguineus or Ornithodoros or Spotted Fever Group Rickettsiosis or Amblyomma) OR TI=(Ticks or Tick-Borne Diseases or Tick Bites or Anaplasmosis or Babesiosis or Colorado Tick Fever or Colorado Tick Fever Virus or Ehrlichiosis or Relapsing Fever or Ixodidae or Lyme Disease or Encephalitis Viruses, Tick-Borne or Encephalitis, Tick-Borne or Rickettsia Infections or rocky mountain spotted fever or Ornithodoros or Argasidae or Tularemia or Hemorrhagic Fever, Crimean or Hemorrhagic Fevers, Viral or Argas or Dermacentor or Haemaphysalis longicornis or Ixodes or Rhipicephalus sanguineus or Ornithodoros or Spotted Fever Group Rickettsiosis or Amblyomma)) AND (AB=(Germany) OR TI=(Germany))
