# Supplementary material for: Risk Factors for Tick‐Borne Diseases in Germany: A Scoping Review
Source: Zoonoses Public Health. 2026 Apr 7;73(4):297–313. doi: 10.1111/zph.70060 (PMC13144437; doi:10.1111/zph.70060)
Supplement: Supplementary file 2 — Table S1: Results of included entomological studies. [file ZPH-73-297-s001.docx]

**Supplementary Material S2**

**Table S1:** Results of included entomological studies

| Authors | Study Period | Location | Measured Outcome | Study population (number and age) | Research design | Key findings | Risk Indicator |
| --- | --- | --- | --- | --- | --- | --- | --- |
| Gethmann et al., 2020 | 2009–2010 | Baden-Wuerttemberg, Bavaria, Saarland, Mecklenburg-Western Pomerania, Lower Saxony, Thuringia and North Rhine-Westphalia | Tick Population of *Ixodes ricinus* | Ticks from 375 flagging | Entomological study | Higher tick abundance was associated with mild air (19–23 °C) and ground (13–15 °C) temperatures, high humidity (35–95%), and June. Forest habitats had significantly more ticks, except adult males. | Humidity, Season, Temperature, Vegetation |
| Hauck et al., 2020 | 2017–2018 (Apr–Oct) | Hanover | Tick Population of *Ixodes ricinus* | 1,770 ticks (2017) and 1,866 ticks (2018) | Entomological study | Abundance was highest in mixed forests, with activity peaking May–June. | Season, Vegetation |
| Raileanu et al., 2022 | 2020 (May–Nov) | German Baltic Coast | Tick Population and PCR for Pathogens of *Ixodes ricinus, D. reticulatus, Haemaphysalis concinna* | 1174 ticks collected: 760 Ixodes ricinus, 326 D. reticulatus, 88 Haemaphysalis concinna | Entomological study | Ixodes ricinus activity peaked May–June (77.2%), D. reticulatus in May (49.1%) and October (26.7%), and Haemaphysalis concinna in June (71.6%). | Season |
| Richter & Matuschka, 2011 | 2006 (May–Oct) & 2007 (Mar–Oct) | Southern Germany | Lyme-borreliosis Diagnosis via PCR | Questing ticks | Entomological study | Tick abundance was highest in unmodified vegetation and lowest in pastures. Nymphs from meadows and fallow land had more LB (27% and 23%) compared to pastures (7%). Similarly in adult ticks, 23% on fallow land and 6% on the pasture had LB. | Vegetation |
| Blazejak et al., 2017 | 2015 (Apr–Oct) | Hanover | Rickettsiales Diagnosis via qPCR | 2,100 ticks | Entomological study | 3.8% were infected with A. phagocytophilum, and 50.8% with Rickettsia spp.. For A. phagocytophilum there was a peak in July (12.0%) and in forests. Rickettsia spp. peaked April–June and the highest prevalence was in “Misburger Wald” (64.3%). Prevalence of Rickettsia spp. increased from 33.3% (2005) to 50.8% (2015). | Season, Vegetation, Year |
